# Supplementary material for: Correlation between Coronary Artery Calcium- and Different Cardiovascular Risk Score-Based Methods for the Estimation of Vascular Age in Caucasian Patients
Source: J Clin Med. 2022 Feb 19;11(4):1111. doi: 10.3390/jcm11041111 (PMC8877766; doi:10.3390/jcm11041111)
Supplement: Supplementary file 1 [file jcm-11-01111-s001.zip › jcm-1534445-supplementary.pdf]

Table S1. Description of vascular age in patients with and without significant coronary artery stenosis.

	With significant stenosis	Without significant stenosis	p-value
Number	27	214	-
Chronological age (years)	61.3 [52.0 – 68.3]	55.8 [47.8 – 66.1]	0.26
CACS vascular age (years)	80.0 [65.0 – 94.2]	39.1 [39.1 – 68.4]	***<0.001***
FRS vascular age (years)	80.0 [64.0 – 84.0]	68.0 [55.0 – 82.0]	***0.03***
SCORE vascular age (years)	70.0 [57.0 – 78.0]	63.0 [52.0 – 75.0]	0.14

The chronological and risk-derived vascular ages were compared between patients with significant stenosis and those without severe coronary disease using Mann-Whitney U-test.

CACS, coronary artery calcium score; Framingham Risk Score, FRS; SCORE, Systematic COronary Risk Evaluation.

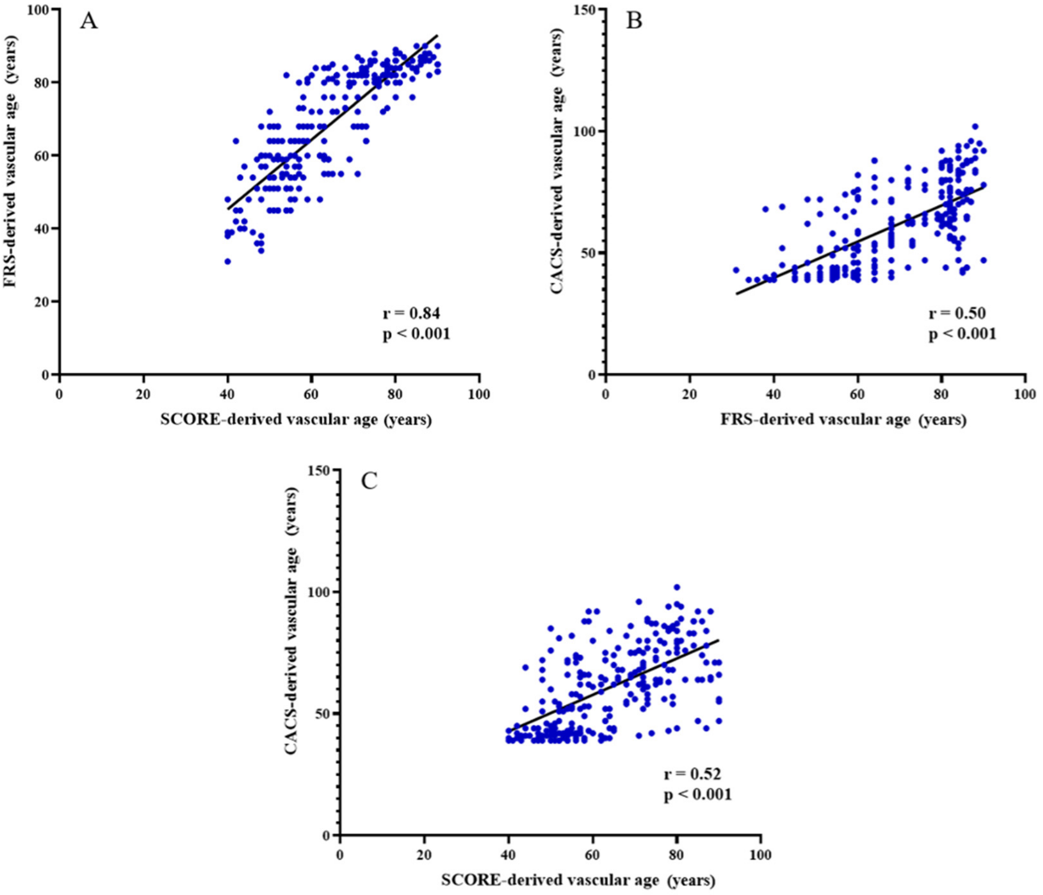

Figure S1. Pearson's correlation coefficient calculated for the pairwise comparison of vascular age derivates. Correlation of different score-based vascular ages as measured by Framingham risk score (FRS) vs. Systematic COronary Risk Evaluation (SCORE) (A), FRS vs. Coronary Artery Calcium Score (CACS) (B), and SCORE vs. CACS (C). Although all comparison combinations showed significant correlations (p<0.001), CACS-derived vascular age showed only moderate correlation with FRS (r=0.50) and SCORE based vascular age (r=0.52), while FRS and SCORE derivates strongly correlated (r=0.84).
